# A comparison of bronchial blocker under artificial pneumothorax and double-lumen endobronchial tube for lung isolation in thoracoscopic enucleation of oesophageal leiomyoma

**DOI:** 10.1186/s13019-021-01707-4

**Published:** 2021-10-31

**Authors:** Luo Zhao, Xue Zhang, Chao Gao, Jia He, Zhijun Han, Li Li

**Affiliations:** 1 Department of Thoracic Surgery, Peking Union Medical College Hospital, CAMS & PUMC, No. 1 Shuaifuyuan, Dongcheng District, Beijing, 100730 China; 2 Department of Anaesthesiology, Peking Union Medical College Hospital, CAMS & PUMC, Beijing, 100730 China

**Keywords:** Oesophageal leiomyoma, Enucleation, Video-assisted thoracoscopy, Artificial pneumothorax, Bronchial blocker

## Abstract

**Background:**

Oesophageal leiomyomas are one of the most common benign oesophageal tumours. This retrospective, observational study summarized and compared the clinical outcomes of thoracoscopic enucleation of oesophageal leiomyoma between single-lumen endotracheal intubation with a bronchial blocker and double-lumen endotracheal intubation.

**Methods:**

A total of 36 patients who underwent thoracoscopic enucleation of oesophageal leiomyoma at Peking Union Medical College Hospital between 2014 and 2020 were retrospectively analysed. Fifteen patients received single-lumen endotracheal intubation combined with a right bronchial blocker (SLT-B group), and twenty-one patients received double-lumen endotracheal intubation (DLT group). Clinical data, surgical variables, and postoperative complications were analysed and compared.

**Results:**

The average tumour size in all patients was 4.3 ± 2.0 cm. The average tumour size among symptomatic patients was significantly larger than that among asymptomatic patients (5.1 ± 2.0 cm vs 3.7 ± 1.7 cm, P < 0.05). Patients in the SLT-B group had a significantly larger average tumour size than patients in the DLT group (5.4 ± 2.1 cm vs 3.5 ± 1.4 cm, P < 0.05). The SLT-B group had a significantly shorter operation time and shorter total hospital stay than the DLT group. No mucosal injury, conversion to thoracotomy, or other operative complications occurred in the SLT-B group. In the follow-up, no recurrence, dysphagia, or regurgitation was found in any of the patients.

**Conclusions:**

Compared with traditional double-lumen intubation, artificial pneumothorax-assisted single-lumen endotracheal intubation combined with a bronchial blocker for thoracoscopic oesophageal leiomyoma enucleation can achieve complete removal of larger tumours, with fewer complications and shorter hospital stays.

## Introduction

Oesophageal leiomyomas are the most common benign oesophageal tumours, accounting for approximately 70%—80% of oesophageal submucosal tumours [1]. In recent years, video-assisted thoracoscopic enucleation of oesophageal leiomyoma has been shown to have better postoperative recovery than the thoracotomy approach [2]. Compared with double-lumen endotracheal intubation anaesthesia, single-lumen endotracheal intubation combined with a bronchial blocker under artificial pneumothorax is an improved lung separation technology. Artificial pneumothorax achieves better lung collapse and surgical field exposure and decreases blood loss, which are more convenient for tumour enucleation with less morbidity [3]. This study retrospectively analysed a total of 36 patients diagnosed with oesophageal leiomyoma undergoing thoracoscopic surgery at Peking Union Medical College Hospital from 2014 to 2020. We present surgical outcomes of video-assisted thoracoscopic enucleation of oesophageal leiomyoma and evaluate the advantages of single-lumen endotracheal intubation combined with a bronchial blocker under artificial pneumothorax compared with traditional double-lumen endotracheal intubation.

## Methods

A total of 36 patients who underwent thoracoscopic enucleation of oesophageal leiomyoma by one surgeon (Dr. Li) at Peking Union Medical College Hospital from 2014 to 2020 were retrospectively selected. All patients were diagnosed with oesophageal leiomyoma by postoperative pathology, and the tumour location was evaluated by gastroscopy, upper gastrointestinal angiography and enhanced computed tomography of the chest and abdomen. Fifteen patients received single-lumen endotracheal intubation combined with a right bronchial blocker under artificial pneumothorax (the SLT-B group, September 2017 to December 2020), and twenty-one patients received traditional double-lumen endotracheal intubation (the DLT group, January 2014 to August 2017). The independent medical ethical committee of the Peking Union Medical College Hospital approved this study (IRB number S-K1717) and all the patients signed extensive informed consent forms.

### Anaesthetic and surgical procedures

All patients underwent a right thoracoscopic side approach, and a nasogastric tube was placed before the operation. General anaesthesia was induced with midazolam 0.05 mg/kg, propofol 2 mg/kg, fentanyl 2ug/kg, rocuronium 0.6–0.9 mg/kg, followed by endotracheal intubation. For all patients, intubation was performed by an experienced attending anaesthesiologist. Anaesthesia sedation was maintained with sevoflurane to keep bispectral index within 40–60. Fentanyl and rocuronium were infused to maintain analgesia and muscular blockade, respectively, as needed.

In the SLT-B group, a 7.0 mm or 7.5 mm internal diameter endotracheal tube was inserted using direct laryngoscope under direction of a stylet. Then, a bronchial blocker (Haisheng Medical Device Corp., Zhejiang, CN) was inserted into the right main bronchus under the guidance of fiberoptic bronchoscope, and the cuff of blocker was placed just below the tracheal carina. Artificial pneumothorax was created by CO2 insufflation with a pressure of 8 mmHg. When surgery began, the cuff of blocker was deflated and ventilation was stopped until the right lung collapsed. One lung ventilation was achieved by inflating the cuff of blocker during surgery, and the airway pressure was maintained under 30 cmH_2_O. In the DLT group, a 35 Fr or 37 Fr left double-lumen endobronchial tube was inserted using direct laryngoscope under direction of a stylet. Tube location was checked using fiberoptic bronchoscope to make sure that the bronchial cuff was just below the tracheal carina in the left main bronchus but before the takeoff of any lobar bronchi. Flow to the right lung was stopped by clamping the tracheal lumen while the bronchial cuff was inflated. The airway pressure was also maintained under 30 cmH_2_O.

The patient was arranged in a left semi-prone position inclined 45 degrees. In the operation, after the tumour was detected, the muscular layer of the oesophagus was opened by a coagulating hook. The tumour was separated from the muscular layer, carefully protecting the mucosa from injuries. After tumour enucleation, the muscular layer and the parietal pleura were closed using continuous 3-0 V-loc stitches. The resected tumour was placed in a bag and removed from the chest. Air inflation by a nasogastric tube was used to confirm whether there was oesophageal mucosal injury. If mucosal injury was found, the oesophageal mucosa was sutured by interrupted absorbable 3-0 stitches. A 28-French thoracic drain was left in the right chest. If no oesophageal mucosa injury was found during the operation, the nasogastric tube was removed by the first day after the operation, and the oral diet was resumed the second day. If mucosal injury was found and repaired during the operation, patients received nothing by mouth with total enteral nutrition for 7 days, oesophagography was performed to confirm mucosal integrity, and the nasogastric tube was removed after confirmation. The thoracic drainage tube was removed after drainage was less than 100 ml and there was no air leak after oral diet feeding.

### Variables collected

We collected data on the baseline characteristics and surgical and postoperative characteristics in this study.

### Statistical analysis

Analysis was performed using Statistical Product and Service Solutions 22.0 statistical software (IBM Corp., Armonk, NY, USA). The continuous variables are expressed as the means (x ± s) or medians (P25, P75). We use Kolmogorov–Smirnov test to determine the distribution of the parameters. The differences between variables were analyzed using the Student’s t test with normal distribution, or the Mann–Whitney U test if not. The chi-squared test was used to compare the frequencies of the categorical variables data. P value < 0.05 were considered significant.

## Results

Patient characteristics are shown in Table 1. There were 23 men and 13 women, with an average age of 43.7 ± 10.6 years. The majority of the tumours arose in the middle (19/36 [52.8%]). Clinical symptoms were found in 16 patients, and 20 (55.6%) patients were asymptomatic, with their tumours discovered incidentally by routine physical examination. The most common symptom that patients reported was dysphagia (10/36 [27.8%]). Most patients had chronic symptoms persisting for months to years before surgical management. The average tumour size in all patients was 4.3 ± 2.0 cm. Among symptomatic patients, the average tumour size was 5.1 ± 2.0 cm, compared with an average tumour size of 3.7 ± 1.7 cm in asymptomatic patients (P = 0.035).

**Table 1:** Baseline characteristics

Variables	Total (N = 36)	SLT-B group (N = 15)	DLT group (N = 21)	P value*
Age (years)	43.7 ± 10.6	45.2 ± 8.9	42.6 ± 11.7	0.469
Sex				0.681
Male	23 (63.9%)	9	14	
Female	13 (36.1%)	6	7	
Tumour location				0.803
Upper	8 (22.2%)	4	4	
Middle	19 (52.8%)	7	12	
Lower	9 (25%)	4	5	
Presenting symptoms				0.923
Asymptomatic	20 (55.6%)	8	12	
Dysphagia	10 (27.8%)	5	5	
Regurgitation	3 (8.3%)	1	2	
Chest discomfort	3 (8.3%)	1	2	
Time to surgery (months)	2 (2, 7.5)	2 (2, 3)	3 (1.5, 9.5)	0.334
Tumour size (cm)				
All patients	4.3 ± 2.0	5.4 ± 2.1	3.5 ± 1.4	0.004
Patients without symptoms	3.7 ± 1.7			0.035**
Patients with symptoms	5.1 ± 2.0			

*Comparison between the SLT-B group and DLT group, **comparison between patients without symptoms and patients with symptoms

Fifteen patients were assigned to the SLT-B group, and twenty-one patients were assigned to the DLT group. No significant differences were observed between the two groups in age, sex, or tumour location. Patients in the SLT-B group had a significantly larger average tumour size than patients in the DLT group (5.4 ± 2.1 cm vs 3.5 ± 1.4 cm, P = 0.004). Surgical and postoperative characteristics are presented in Table 2. The SLT-B group had a significantly shorter operation time, shorter duration of chest tube and total hospital stay than the DLT group. No mucosal injury, conversion to thoracotomy, or other operative complications occurred in the SLT-B group. Two cases of mucosal injury occurred in the DLT group and were repaired during the operation. The patients were discharged from the hospital after conservative treatment. The period of follow-up was 6 months to 6 years. In the follow-up, no recurrence, dysphagia, or regurgitation was found in any of the patients.

**Table 2:** Surgical and postoperative characteristics

Variables	Total (N = 36)	SLT-B group (N = 15)	DLT group (N = 21)	P value*
Operation time, min	107.5 (90, 135)	100 (75, 110)	120 (97.5, 150)	0.030
Estimated blood loss (ml)	27.5 (20, 50)	25 (20, 50)	30 (20, 50)	0.812
Mucosal injury	2 (5.6%)	0	2 (9.5%)	0.219
Postoperative complication	0	0	0	NA
Duration of chest tube	4.0 ± 2.2	2.5 ± 0.5	5.0 ± 2.4	< 0.001
Total hospital stay (days)	5.3 ± 2.3	3.9 ± 1.3	6.2 ± 2.4	0.001

*Compared between SLT-B group and DLT group

## Discussion

Oesophageal leiomyomas are the most common benign tumours of the oesophagus. It has been reported that the disease mainly occurs in the middle part of the oesophagus, followed by the lower and upper parts of the oesophagus [4]. The tumour grows slowly, and small oesophageal leiomyomas are usually asymptomatic. With the growth of the tumour, patients may present acid regurgitation, chest pain, dysphagia and dyspepsia. With the development of gastroscopy and ultrasonic gastroscopy technology, an increasing number of asymptomatic small oesophageal leiomyomas have been detected and treated [5]. However, in symptomatic patients, oesophageal leiomyomas are usually larger, which increases the difficulty in treatment.

At present, surgical treatment is the preferred choice for oesophageal leiomyoma, and enucleation is widely accepted as an adequate treatment [6]. However, surgical indications for oesophageal leiomyoma are still controversial. Oesophageal leiomyoma may grow gradually, which may lead to surrounding. It is suggested that once diagnosed, surgical treatment should be performed regardless of symptoms [7]. Codipilly et al. summarized the clinical data of 105 patients with submucosal tumours and found that small leiomyomas grew very slowly, with an average growth rate of 0.5 mm every 6 years [8]. In our study, the average size of the tumour was 4.3 ± 2.0 cm, and the proportion of symptomatic patients was 44.4% (16/36). Therefore, we indicate surgical enucleation for patients with symptomatic or larger oesophageal leiomyomas (larger than 2 cm), while patients with smaller and asymptomatic oesophageal leiomyomas could be observed and then treated when they were obviously enlarged or had the possibility of malignancy.

Surgery is the therapy of choice. Compared with traditional thoracotomy, video-assisted thoracoscopic surgery has the advantages of minimal scarring, less pain, better postoperative respiratory function with fewer perioperative respiratory complications, and an enhanced fast recovery. It is generally considered that a right thoracic approach is used for middle and upper oesophageal tumours, and a left thoracic approach is used for lower oesophageal tumours [9]. In our study, all patients completed tumour resection through the right thoracic approach, indicating that the right thoracic approach can complete all operations. Thoracoscopic surgery in the prone position has been adopted in the majority of minimally invasive surgeries for oesophagus because it is easy to mobilize the oesophagus [10]. Single-lumen endotracheal intubation combined with a bronchial blocker under artificial pneumothorax has the following advantages: 1. Single-lumen endotracheal intubation is relatively simple, and a bronchial blocker can provide an effective seal of the bronchus with minimal trauma to achieve single-lung ventilation. 2. Single-lumen endotracheal intubation causes less damage to the airway mucosa and respiratory tract, which can reduce postoperative pharyngeal discomfort or pain. 3. Artificial pneumothorax (8 mmHg) can obtain good lung collapse, cause capillaries to collapse and reduce bleeding, maintain a better surgical field and reduce side injury. In our study, complete tumour enucleation was achieved in patients in the SLT-B group, with a larger average tumour size and no mucosal damage or other complications and a shorter postoperative hospital stay than patients in the DLT group. Thoracoscopic operation in the prone position with artificial pneumothorax with CO_2_ insufflation may add merits to the conventional decubitus position.

Oesophageal mucosal injury is the most common complication, especially when the tumour is large and close to the oesophageal mucosa. According to the author's experience, blunt dissection should be used to dissect the tumour, and energy instruments should be avoided near the oesophageal mucosa side to prevent intraoperative oesophageal mucosal injury. After the enucleation of the tumour, the mucosal wound should be carefully examined. Absorbable sutures should be used to repair the injured oesophageal mucosa. For large mucosal injuries, the mediastinal pleura should be used to cover the injured part after repair, and the nasogastric tube should be reserved. After 7 days of fasting, upper oesophagography should be performed to determine whether the injury had healed.

According to previous research, carbon dioxide artificial pneumothorax under low pressure (< 8 mmHg) has no significant effect on respiration and circulation [11]. In our study, we did not observe any respiratory or haemodynamic disorders perioperatively. Another disadvantage is the inability of continuous sucking of the operative field, which may cause difficulty in the haemostatic process and increase the operation time. In our study, however, the operation time was significantly shorter in the SLT-B group. Therefore, we believe that this shortcoming can be overcome through more experience with this surgical technique.

Nevertheless, several limitations in our study are noted. First, the nature of retrospective analysis is a limitation, such as patient selection bias may exist even though baseline characteristics were comparable. Second, the SLT-B group has only been applied since 2017, thus, more experience with surgical technique may contribute to a better outcome in this group. In addition, the strength of this study is weak that this cohort consists of a small sample size. Thus, larger-scale studies, especially homogeneous multicentre collaboration, are needed.

## Conclusions

Our results provide the outcomes of thoracoscopic enucleation of oesophageal leiomyoma in a single institution. Application of single-lumen endotracheal intubation combined with bronchial blocker under artificial pneumothorax is recommended as a feasible and safe procedure.

## Data Availability

The datasets supporting the conclusions of this article are included within the article and its additional files.

## Abbreviations

SLT-B: Single-lumen endotracheal intubation combined with right bronchial occlusion
DLT: Double-lumen endotracheal intubation
